# Phenotyping Pediatric Long COVID: Symptom Clusters from a Longitudinal Multicenter Italian Cohort

**DOI:** 10.3390/children13020279

**Published:** 2026-02-18

**Authors:** Susanna Maria Roberta Esposito, Giuseppe Maglietta, Beatrice Rita Campana, Valentina Fainardi, Marco Poeta, Stefania Zampogna, Claudia Colomba, Agnese Suppiej, Fabio Cardinale, Samantha Bosis, Elio Castagnola, Fabio Midulla, Carlo Giaquinto, Paola Giordano, Giacomo Biasucci, Francesco Nunziata, Roberto Grandinetti, Anna Condemi, Giuseppe Raiola, Alfredo Guarino, Francesca Diodati, Caterina Caminiti

**Affiliations:** 1 Pediatric Clinic, Department of Medicine and Surgery, University Hospital of Parma, 43126 Parma, Italy; susannamariaroberta.esposito@unipr.it (S.M.R.E.); beatricerita.campana@unipr.it (B.R.C.); valentina.fainardi@unipr.it (V.F.); roberto.grandinetti@unipr.it (R.G.); 2 Clinical and Epidemiological Research Unit, University Hospital of Parma, 43126 Parma, Italy; fdiodati@ao.pr.it (F.D.); ccaminiti@ao.pr.it (C.C.); 3 Pediatric Infectious Disease Unit, Department of Maternal and Child Health, University Hospital “Federico II”, 80138 Naples, Italy; marco.poeta@unina.it (M.P.); alfredo.guarino@unina.it (A.G.); 4 Department Pediatrics, Hospital of Crotone, 88900 Crotone, Italy; stefania.zampogna@asp.crotone.it; 5 Division of Pediatric Infectious Diseases, “G. Di Cristina” Hospital, ARNAS Civico Di Cristina Benfratelli, University of Palermo, 90128 Palermo, Italy; claudia.colomba@unipa.it; 6 Department of Medical Sciences-Pediatric Section, University of Ferrara, 44121 Ferrara, Italy; agnese.suppiej@unife.it; 7 Complex Operating Unit Paediatrics, Giovanni XXIII Paediatric Hospital, University of Bari, 70124 Bari, Italy; fabio.cardinale@libero.it; 8 Pneumology and Infectious Diseases Unit, Fondazione IRCCS Ca’ Granda Ospedale Maggiore Policlinico, 20122 Milan, Italy; samantha.bosis@policlinico.mi.it; 9 Pediatric Infectious Diseases Unit, IRCCS Istituto Giannina Gaslini, 16147 Genoa, Italy; eliocastagnola@gaslini.org; 10 Department of Maternal, Infantile and Urological Sciences, Sapienza University of Rome, 00161 Rome, Italy; midulla@uniroma1.it; 11 Division of Pediatric Infectious Diseases, Department of Women’s and Children’s Health, University of Padua, 35128 Padua, Italy; carlo.giacquinto@unipd.it; 12 Pediatric Unit, Department of Interdisciplinary Medicine, University of Bari "Aldo Moro", 70124 Bari, Italy; paola.giordano@uniba.it; 13 Pediatrics and Neonatology Unit, Department of Medicine and Surgery, University of Parma, Guglielmo da Saliceto Hospital, 29121 Piacenza, Italy; g.biasucci@ausl.pc.it; 14 General Pediatrics and Immuno-Rheumatology Unit, Santobono-Pausilipon Children’s Hospital, AORN, 80129 Naples, Italy; f.nunziata@santobonopausilipon.it; 15 Department of Health Promotion, Maternal and Infant Care, Internal Medicine and Medical Specialties, University of Palermo, Palermo 90100, Italy; anna.condemi@unipa.it; 16 Department of Pediatrics, “Pugliese-Ciaccio” Hospital, 88100 Catanzaro, Italy; graiola@aocz.it

**Keywords:** COVID-19, SARS-CoV-2, post-acute COVID-19 syndrome, long COVID, pediatrics, child, adolescent, cluster analysis, symptom assessment, symptom cluster

## Abstract

**Highlights:**

**What are the main findings?**

- In a longitudinal cohort of 850 children, cluster analysis identified three age-related trajectories.
- Young children (0–5 years) mainly had respiratory symptoms and higher hospitalization risk, with sequelae often persisting; older children (6–11 years) experienced mild disease and good recovery; adolescents (12–17 years), particularly females, showed more severe acute symptoms and were most affected by long-term neuropsychological sequelae like fatigue and insomnia.

**What are the implications of the main findings?**

- Pediatric PASC management should be age-tailored: vaccination and respiratory follow-up should be reinforced in young children, and neuropsychological support should be prioritized for adolescent girls.
- Future research should confirm these patterns in larger groups of people, observe how they change over time, and combine biological and psychosocial information, as well as potential biomarkers and objective measures (e.g., neurocognitive testing and pulmonary function), to help design prevention and intervention strategies.

**Abstract:**

**Background:** The aim of this study was to identify patient clusters based on acute symptom profiles and individual characteristics most likely to develop pediatric post-acute sequelae of SARS-CoV-2 infection (PASC), as well as clusters among patients with PASC based on post-acute sequelae and associated characteristics. **Methods:** This multicenter cohort study in 12 Italian pediatric units enrolled patients aged 0–17 years within three months of a laboratory-confirmed SARS-CoV-2 infection. Participants who completed at least two surveys developed by the ISARIC over one year were analyzed. PASC was defined per WHO criteria. Multiple Correspondence Analysis and Hierarchical Clustering were performed. **Results:** Of 1137 children enrolled, 850 (76%) completed at least two surveys. The most prevalent age group was older children (6–11 years) (46%); adolescents (12–17) and young children (0–5) were numerically similar. Males were more represented (51.9%), except for the adolescent group (45.1%). PASC occurred in 32.8% of participants, with the distribution of sequelae types varying by age. Clustering in COVID-19 cases identified three clusters: young children mainly presented with respiratory symptoms and with a higher risk of hospitalization, while older children were spared in both acute and post-acute phases. Adolescents, particularly females, reported more pronounced acute symptoms and developed PASC more frequently. Clustering analysis of cases with PASC identified three clusters, confirming these age-related patterns. Young children still exhibited respiratory sequelae, and older children confirmed good recovery with minimal complications, while adolescents, especially females, remained the most affected subgroup, reporting persistent neuropsychological sequelae such as fatigue and insomnia. **Conclusions:** Findings support age-tailored follow-up, emphasizing respiratory monitoring for young children and targeted neuropsychological care for adolescents, particularly girls.

## 1. Introduction

Although the acute phase of the COVID-19 pandemic has subsided, its long-term effects persist in the form of ongoing signs and symptoms well beyond the resolution of the initial infection [1,2]. This condition—commonly referred to as long COVID, or post-acute sequelae of SARS-CoV-2 infection (PASC)—is now recognized as a significant global cause of disability across all age groups [3]. Despite this, most research efforts aimed at understanding and treating PASC have focused primarily on adults [3,4]. This is largely due to the assumption that COVID-19 in children generally results in mild or asymptomatic disease with limited complications, although growing evidence suggests that young people can experience persistent health problems following SARS-CoV-2 infection, even when the acute disease was mild or unnoticed [5,6,7,8]. The burden of pediatric PASC is substantial—current estimates suggest that up to 20% of children with a history of COVID-19 develop long-term symptoms [5].

Underestimating pediatric PASC may have serious consequences, as post-acute sequelae in children have been associated with impairments across physical, neurocognitive, psychosocial, and quality-of-life domains [9,10]. Importantly, the long-term impact of COVID-19 in children can differ from adults. Recognizing this, the World Health Organization (WHO) issued a specific clinical case definition for long COVID in children and adolescents [11], underscoring the need for targeted research in this population. Several knowledge gaps continue to impede the effective diagnosis and management of pediatric PASC. First, the condition is difficult to diagnose and identify, as it often presents with a wide range of non-specific and fluctuating symptoms [8,9,12,13]. Furthermore, some healthcare providers remain skeptical about the existence or severity of PASC, particularly in children and adolescents, and may underestimate its physiological and psychological impacts [4,12]. Another significant barrier is our limited understanding of the underlying mechanisms driving pediatric PASC [9,14]. A mechanistic approach—targeting subtypes of disease based on shared pathophysiology—may support tailored care strategies and inform the development of more personalized therapies that go beyond symptom management [13,15]. The current lack of standardized diagnostic tools and clinical management guidelines for children, in contrast to existing protocols for adults, reflects the broader gaps in pediatric-specific knowledge [13].

Addressing these gaps requires a clearer understanding of how symptoms occurring in the acute phase of COVID-19 disease relate to long-term sequelae, and whether they cluster into distinct phenotypes with shared etiologies. Symptom clustering analysis—a method used to identify patterns of co-occurring symptoms—can provide valuable insights into underlying mechanisms and help refine diagnostic pathways [16]. This approach can also guide the development of targeted interventions and support care that is better tailored to affected individuals [17,18].

While symptom-clustering research has been widely applied in adult PASC studies, research in pediatric populations remains limited. Existing studies often suffer from small sample sizes [19,20] and misclassification bias [21] or are restricted to a specific pediatric age [22]. Other studies rely solely on secondary data sources such as electronic health records using machine learning techniques [23,24,25]. In particular, recent large pediatric phenotyping efforts such as the U.S. RECOVER consortium have applied latent class analysis and machine learning approaches to identify distinct long COVID symptom patterns in children and adolescents [21,23,24,25]. However, the study population was not consecutively enrolled but instead selected using stringent eligibility criteria. In addition, younger children (<6 years) were not considered, and COVID-19 positivity did not require laboratory confirmation. To address these limitations, we conducted a longitudinal study based on patient-reported data, including reports from caregivers for younger children. The primary aim of this study was to identify distinct clusters of pediatric patients, based on their symptom profiles and individual characteristics during the acute phase of COVID-19, who are at increased risk of developing PASC. Furthermore, among those who developed PASC, the study sought to characterize symptom-based clusters and their associated patient features in order to inform more precise, targeted clinical interventions.

## 2. Materials and Methods

### 2.1. Study Design, Setting, and Participants

This study expands upon our previous longitudinal investigation within the International Severe Acute Respiratory and Emerging Infection Consortium (ISARIC) pediatric framework [6]. The research was conducted across 12 pediatric units in Northern Italy, with the primary aim of characterizing PASC in the Italian pediatric population. The study was conducted in accordance with the Declaration of Helsinki and was approved by the Area Vasta Emilia Nord (AVEN) Local Ethics Committee on 30 November 2021 (protocol no. 952/2021/OSS/AOUPR).

Full details of the study methodology have been published elsewhere [6].

Briefly, the study enrolled individuals aged 0–17 years within three months of a laboratory-confirmed SARS-CoV-2 infection, including both hospitalized and non-hospitalized cases. Subjects were identified from electronic medical records and the Local Health Information System at the host institutions. Enrolment began in January 2022 and ended in November 2022. Participants (or their caregivers in the case of younger children) completed a standardized questionnaire developed by ISARIC [26], either online or via telephone interviews at three time points: 1–3 months, 3–6 months, and 6–12 months post-infection.

The questionnaire [26] collected data on demographics, clinical history, comorbidities (diagnosis or treatment for a health problems occurring prior to COVID-19 infection and still ongoing), 17 acute symptoms, i.e., symptoms manifesting in the acute phase (within 14 days of infection), hospitalization, COVID-19 vaccination status, and the presence of 26 post-acute sequelae, i.e., problems that were not present before the infection occurring within the past seven days. In addition, at enrolment respondents were asked to indicate their perceived level of recovery using the question: ‘How much do you agree with the following statement? I have fully recovered from my infection’, rated on a 0–10 Likert scale. Responses were categorized into two groups: 0–6 (disagree) and 7–10 (agree).

In accordance with the WHO definition for children and adolescents [11], PASC was defined as symptoms persisting for at least two months, occurring within three months post-infection, and not attributable to another diagnosis. Based on this definition, the analyses in this study focused on the cohort of respondents who completed at least two surveys within a one-year period, allowing us to determine the presence of COVID-19 sequelae.

### 2.2. Statistical Analyses

Descriptive statistics were used to summarize demographic and clinical characteristics. Continuous variables were presented as means and standard deviations (SDs) or medians with interquartile ranges (IQRs), depending on distribution. Categorical variables were reported as frequencies and percentages. Differences in proportions between groups were assessed using the chi-square or non-parametric tests, as appropriate. Data were analyzed according to age group (young children—0–5 years; older children—6–11 years; and adolescents—12–17 years).

To investigate relationships among acute symptoms, post-acute sequelae, and patient characteristics, we employed Multiple Correspondence Analysis (MCA) as a preliminary step to clustering. MCA reduces dimensionality in categorical data and assigns factor scores to both variables and individuals, allowing for visual representation in a two-dimensional space. The first two orthogonal dimensions captured the greatest variance in the dataset. Active variable contributions to each dimension were quantified and displayed in bar plots. Supplementary qualitative variables were added only to help in the interpretation, without changing the core analysis.

Hierarchical Clustering on Principal Components (HCPC) was then applied to the MCA individual coordinates, grouping patients into clusters based on their proximity in this reduced-dimensional space and classifying participants into symptom clusters based on shared characteristics. All statistical analyses were performed using R Statistical Software (version 4.3.0).

## 3. Results

### 3.1. Clinical and Demographic Characteristics

The overall study enrolled 1137 children diagnosed with SARS-CoV-2 infection who had completed the survey at least once. Of these, 1135 completed the first survey, 859 the second and 732 the third. This analysis therefore included 850 subjects who could be evaluated for the outcomes of interest, i.e., those with at least two surveys, representing 76% of the total. Their baseline demographic and clinical characteristics at enrolment, including the frequency of the acute symptoms, are shown in Table 1.

Overall, the mean age was 7.8 (SD 4.2); the most prevalent age group consisted of older children (46%), while adolescents (26%) and young children (28%) were numerically similar. The male sex was more represented (51.9%), except for the adolescent group, where females constituted the majority (54.9%). Over one-third of participants (36.8%) reported having at least one comorbidity, though the distribution varied by age group, with a higher prevalence among adolescents (44.2%). Approximately one quarter of subjects (210/850, 24.7%) were vaccinated against COVID-19, exhibiting different distribution by age: 56.3% of adolescents, 20.2% of older children and only 2.1% of young children. The proportion of patients hospitalized during the acute phase of COVID-19 infection also varied across age groups, with the highest observed in young children (18.7%), against 1–2% for the other two age groups.

### 3.2. Frequency and Distribution of Acute Symptoms

The most common symptoms reported as absent before COVID-19 and appearing in the first 14 days of the disease were fever ≥38 °C (49%), runny nose (46%), cough (40%), headache (38%) and fatigue (27%). Symptom distribution varied significantly across age groups (Pearson’s χ^2^ test, *p* < 0.001), showing a clear gradient: fever was more frequent in young children (60% vs. 44% in adolescents), whereas headache (53% adolescents vs. 9% young children), sore throat (38% adolescents vs. 20% young children), and muscle pain (30% adolescents vs. 6% young children) became progressively more common with increasing age.

Overall, in the first survey, 84% of participants reported agreement (score 7–10) with the statement ‘I have fully recovered from my infection’, with an almost identical distribution across the three age groups.

### 3.3. Occurrence of PASC and Related Sequelae

**Figure 1:**
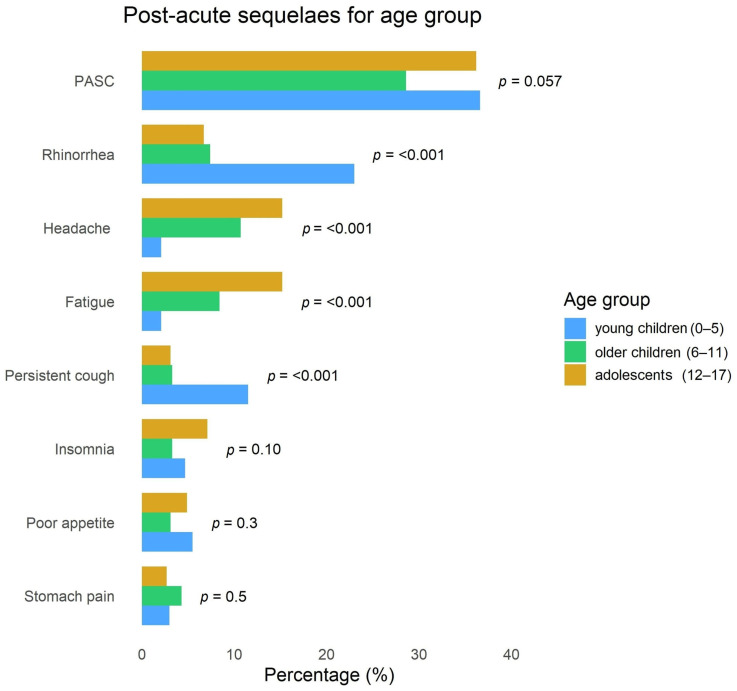
Occurrence of PASC and post-acute sequelae by age groups. Legend: Only post-acute sequelae with a frequency > 3% were included.

Figure 1 depicts the distribution of cases with PASC and post-acute sequelae by age group. Overall, 32.8% (279/850) of the sample experienced PASC, with higher frequencies observed at both ends of the age spectrum (36.2% in adolescents and 36.6% in young children). Distribution of sequela types appeared to vary by age group. Specifically, the most common long-lasting symptoms were respiratory (23% rhinorrhea and 12% persistent cough) in young children, and neurological (15% headache, 15% fatigue and 7% insomnia) in older children.

### 3.4. Clustering of Cases with COVID-19 Infection

**Figure 2:**
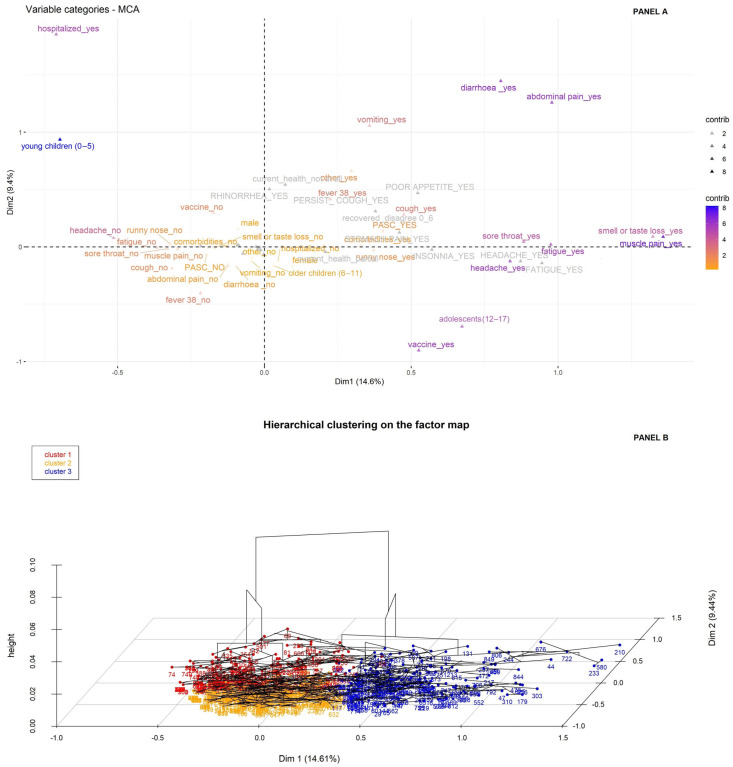
Clustering among all enrolled patients. Legend: Bi-dimensional plot of Multiple Correspondence Analysis (MCA, panel A) and three-dimensional plot graph from Hierarchical Clustering on Principal Components (HCPC) analysis (panel B). Panel A displays the first dimension along the x-axis and the second dimension along the y-axis. The distance from the barycenter, the proximity to the orthogonal axes and the contribution values (from low values in orange to high values in blue) identify the variables most closely associated with the two dimensions. The variables in gray were analyzed only as supplementary quality variables for descriptive purposes. Panel B displays the MCA plot in which patients are grouped according to their assigned cluster distinguished by color. The third dimension shows the cluster dendrogram.

Our first objective was to identify clusters of patients who are more likely to develop PASC, based on symptom profiles and individual characteristics during the acute phase. To this end, we initially conducted an MCA, the results of which are presented in Figure 2 panel A. The analysis was carried out considering 18 active variables (pertaining to presence/absence of PASC, 12 acute symptoms, sex, age, comorbidities, vaccination status, hospitalization), and the remaining variables (seven post-acute sequelae and the perceived level of recovery) were used only as supplementary qualitative variables (colored in gray in Figure 2 panel A).

This analysis showed that the first two components explained about 24% of the overall variability, in which the highest contribution for the first dimension (X axis, 14.6%) was given by the symptoms muscle pain, loss of smell/taste, sore throat, headache and fatigue, whereas the second dimension (Y axis, 9.4%) was prevalently described by age, vaccination status and hospitalization (Figure S1)

Based on this map, the HCPC analysis identified three clusters (Figure 2 panel B and Table S1):

- Cluster 1 consisted of young children (v-test = 25.40) who were predominantly hospitalized (v-test = 10.12) and unvaccinated against COVID-19 (v-test = 11.45). This cluster presented fever during the acute phase (v-test = 5.61), and rhinorrhea (v-test = 6.12) and persistent cough (v-test = 3.94) as post-acute sequelae.
- Cluster 2 comprised older children (v-test = 13.14) who reported no acute symptoms and who generally did not develop PASC (v-test = 6.58).
- Cluster 3 was characterized by adolescent girls (v-test = 8.63 and 1.98), predominantly vaccinated, with comorbidities (v-test = 4.63 and 4.64). This cluster exhibited acute symptoms including headache (v-test = 16.72), myalgia (v-test = 15.67), fatigue (v-test = 14.25), and anosmia/ageusia (v-test = 10.97) and generally developed PASC (v-test = 5.87) with sequelae including headache, fatigue, and insomnia (v-test = 6.30, 5.96 and 3.23, respectively).

### 3.5. Clustering of Cases with PASC

**Figure 3:**
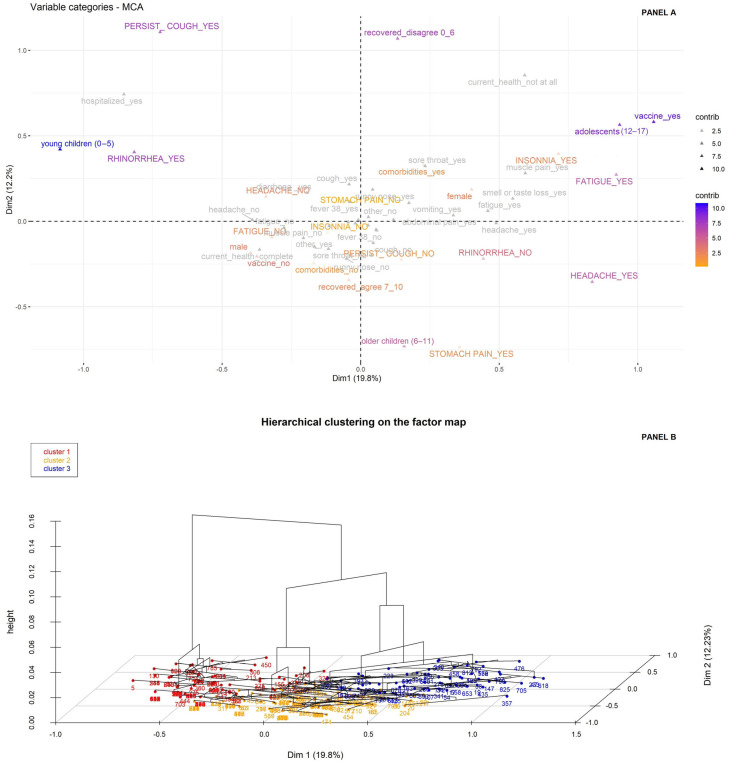
Clustering among patients with PASC. Legend: Bi-dimensional plot of Multiple Correspondence Analysis (MCA, panel A) and three-dimensional plot graph from Hierarchical Clustering on Principal Components (HCPC) analysis (panel B). Panel A displays the first dimension along the x-axis and the second dimension along the y-axis. The distance from the barycenter, the proximity to the orthogonal axes and the contribution values (from low values in orange to high values in blue) identify the variables most closely associated with the two dimensions. The variables in gray were analyzed only as supplementary quality variables for descriptive purposes. Panel B displays the MCA plot in which patients are grouped according to their assigned cluster distinguished by color. The third dimension shows the cluster dendrogram.

To pursue our second objective, namely to identify clusters based on symptoms and associated characteristics among patients with PASC (no. 279), we first performed an MCA considering 11 active variables (6 post-acute sequelae, sex, age, vaccination status, comorbidities, and the perceived level of recovery), and the remaining variables were used only as supplementary qualitative variables (colored in gray in Figure 3 panel A).

This analysis showed that the first two components explained about 32% of the overall variability, in which the highest contribution for the first dimension (19.8%) was given by age group and vaccination status, whereas the second dimension (12.2%) was prevalently described by the perceived level of recovery (Figure S2).

Based on this map, the HCPC analysis identified three clusters (Figure 3 panel B and Table S2):

- Cluster 1 consisted of young children (v-test = 14.56) who were predominantly unvaccinated against COVID-19 (v-test = 6.07). Post-acute phase features included rhinorrhea (v-test = 8.58) and a persistent cough (v-test = 6.64).
- Cluster 2 comprised older children (v-test = 11.71), predominantly male (v-test = 2.83), reporting full recovery (v-test = 5.43). Post-acute sequelae included headache and stomach/abdominal pain (v-test = 3.31 and 3.02).
- Cluster 3 consisted of adolescents (v-test = 11.98), predominantly girls (v-test = 5.51), vaccinated (v-test = 11.55), with perceived incomplete recovery and comorbidities (v-test = 3.46 and 2.10). Post-acute sequelae included insomnia (v-test = 3.73) and fatigue (v-test = 5.19).

## 4. Discussion

Post-acute sequelae of SARS-CoV-2 infection, or long COVID, represent an emerging challenge in pediatric medicine. Unlike the acute phase, which is relatively well characterized, the long-term consequences in children and adolescents remain only partially understood. The condition manifests as a heterogeneous set of physical, cognitive, and emotional symptoms that may co-occur and persist over time, reflecting overlapping biological mechanisms and psychosocial factors. This complexity has hindered the establishment of a universally accepted clinical definition and limited clinicians’ ability to recognize and manage the disorder [27]. Identifying reproducible symptom patterns is therefore crucial to clarify disease mechanisms, improve diagnostic frameworks, and guide the development of tailored interventions [28]. Our study contributes to this effort by applying cluster analysis to a large multicenter Italian pediatric cohort, identifying three age-related symptom clusters, each associated with different probabilities of developing post-acute sequelae.

From a clinical perspective, the identification of age-related clusters at the onset of SARS-CoV-2 infection supports the presence of distinct pediatric trajectories of post-acute outcomes. Young children tend to follow a predominantly respiratory pattern, a phenomenon that may also be influenced by the circulation of multiple respiratory viruses in this age group, whereas vulnerability to PASC appears lowest in older children. In contrast, adolescents represent the group at highest risk for persistent symptoms, even in the presence of vaccination, underscoring the need for age-stratified follow-up and management strategies. It should be pointed out that during the enrolment period COVID-19 vaccination was recommended but not mandatory for minors older than 5 years, and vaccination was performed in young children only if they were considered at high risk.

The clustering analysis restricted to children with PASC further supported the presence of age-specific post-acute trajectories. Young children predominantly showed respiratory sequelae, whereas older children generally exhibited favorable recovery. In contrast, adolescents—particularly females—remained the most affected subgroup, characterized by persistent neuropsychological symptoms. These age- and sex-related patterns are consistent with previous pediatric COVID-19 studies [29,30,31], including one conducted by our group [32]. Beyond confirming existing evidence [19,20,21,22], our approach integrates symptom profiles with demographic characteristics and outcomes, making the findings particularly useful for clinical practice compared with other clustering-based studies. Our findings align in part with those obtained in the RECOVER study, a benchmark work in the field [21,23,24,25]. Specifically, cluster overlapping is represented by adolescents with sequelae of fatigue and insomnia and older children with gastrointestinal sequelae. However, other findings did not overlap, potentially due to differences in objectives, population and methodological approaches.

Another strength of our work lies in the use of the ISARIC tool for data collection. This standardized instrument, widely adopted internationally, ensures consistency and comparability across study sites [33,34]. Developed through collaboration with experts from multiple disciplines and with input from patient and public representatives, including individuals living with long COVID [35], the tool strengthens both the validity and generalizability of our data.

The sample also represents a distinctive feature of this study. We included pediatric individuals of all ages (newborn to 17 years), consecutively identified from healthcare databases, with laboratory-confirmed COVID-19. This approach, unlike that of other comparable studies [19,20,22], which enrolled only children already reporting PASC or recruited from specialized centers, ensured representativeness of the general pediatric population. Notably, only Weakley et al. [19] explicitly required laboratory-confirmed infection, underlining a further strength of our study design.

Another relevant methodological choice concerns the absence of a non-infected control group. This differentiates our work from the RECOVER study [21], which included controls. While such an approach facilitates causal inference by highlighting symptoms more frequent in infected children, it also carries the risk of misclassification, particularly when infection status is uncertain. In RECOVER, for instance, SARS-CoV-2 infection was not always laboratory-confirmed, raising the possibility of incorrect group assignment and attenuation of differences. Conversely, although our design limits attribution of symptoms exclusively to PASC, it eliminates this potential bias.

This study has several limitations. First, approximately one quarter of participants were lost to follow-up. However, baseline characteristics of these individuals were comparable to those of the analyzed cohort, suggesting a low risk of attrition bias. Second, the absence of a concurrent healthy control group limits causal inference and hampers the ability to disentangle post-SARS-CoV-2 sequelae from pandemic-related psychosocial effects. Nonetheless, the consistency of findings across outcomes and subgroups supports the robustness of our results.

Additionally, the relatively low proportion of variance explained by the first two dimensions (approximately 24–32%) reflects the intrinsic complexity and heterogeneity of long COVID symptomatology, with some variability likely remaining unaccounted for. The follow-up period was limited to a maximum of 12 months, precluding evaluation of temporal changes and stability of symptom clusters over longer time frames [16]. Furthermore, we did not incorporate validated instruments—such as quality-of-life or functional outcome scales—as external anchors or correlates of the identified clusters, as done in the RECOVER study [21]. While such measures could have enhanced clinical interpretability, their inclusion would likely have reduced participation during the pandemic. The identified clusters were also largely characterized by non-specific symptoms, with loss of smell and taste being the only features specific to COVID-19. Although disease-specific clusters would be more informative, they remain difficult to delineate given the heterogeneous and multisystem nature of PASC [28]. Finally, as with all self-reported data, recall bias cannot be excluded.

Moreover, the follow-up covered a maximum of 12 months, preventing evaluation of temporal changes in symptom clusters over time [16]. Second, we did not include validated scales (e.g., quality of life measures) as anchors or correlates of clusters, as done in RECOVER [21]; however, additional questionnaires would likely have reduced participation during the pandemic. Third, our clusters predominantly included non-specific symptoms, with only smell and taste loss representing specific COVID-19 features. Although disease-specific clusters would be more informative, they remain challenging to define given the heterogeneous nature of PASC [28]. Finally, as with all self-reported data, recall bias cannot be excluded.

Despite these limitations, our study highlights the need for age-tailored clinical strategies, reinforcing vaccination and respiratory follow-up for young children while prioritizing neuropsychological support and symptom management programs for adolescent girls. These findings underscore the value of cluster analysis for risk stratification and tailored management. Future research should validate these profiles in larger populations, track their evolution over time, and integrate potential biomarkers or objective measures (e.g., neurocognitive testing, pulmonary function) to guide prevention and intervention strategies.

## 5. Conclusions

Our study highlights the need for age-tailored clinical strategies, reinforcing vaccination and respiratory follow-up for young children, while prioritizing neuropsychological support and symptom management programs for adolescent girls. These findings underscore the value of cluster analysis for risk stratification and tailored management. Future research should validate these profiles in larger populations, track their evolution over time, and integrate biological and psychosocial data to guide prevention and intervention strategies.

## Figures and Tables

**Table 1:** Population characteristics and prevalence of acute symptoms by age group.

Variable	Overall N = 850 (100%) ^3^	Adolescents ^1^ N = 224 (26.3%) ^3^	Older Children ^1^ N = 391 (46%) ^3^	Young Children ^1^ N = 235 (27.7%) ^3^	*p*-Value ^2^
Age, mean (SD), y	7.8 (4.2)	13.0 (2.1)	8.1 (1.4)	2.4 (1.7)	
Sex, female	409 (48.1%)	123 (54.9%)	183 (46.8%)	103 (43.8%)	0.046
Comorbidities: ^4,5^	313 (36.8%)	99 (44.2%)	148 (37.9%)	66 (28.1%)	0.001
Vaccinated ^4^	210 (24.7%)	126 (56.3%)	79 (20.2%)	5 (2.1%)	<0.001
Hospitalized ^4^	53 (6.2%)	5 (2.2%)	4 (1.0%)	44 (18.7%)	<0.001
Acute symptoms ^6^					
Fever ≥38	419 (49.3%)	99 (44.2%)	178 (45.5%)	142 (60.4%)	<0.001
Runny nose	394 (46.4%)	124 (55.4%)	168 (43.0%)	102 (43.4%)	0.007
Cough	339 (39.9%)	96 (42.9%)	135 (34.5%)	108 (46.0%)	0.010
Headache	323 (38.0%)	119 (53.1%)	182 (46.5%)	22 (9.4%)	<0.001
Fatigue	232 (27.3%)	89 (39.7%)	120 (30.7%)	23 (9.8%)	<0.001
Sore throat	231 (27.2%)	86 (38.4%)	98 (25.1%)	47 (20.0%)	<0.001
Muscle pain	152 (17.9%)	67 (29.9%)	71 (18.2%)	14 (6.0%)	<0.001
Loss of smell/taste	97 (11.4%)	45 (20.1%)	47 (12.0%)	5 (2.1%)	<0.001
Abdominal pain	95 (11.2%)	24 (10.7%)	50 (12.8%)	21 (8.9%)	0.30
Vomiting	91 (10.7%)	25 (11.2%)	33 (8.4%)	33 (14.0%)	0.087
Diarrhea	90 (10.6%)	23 (10.3%)	38 (9.7%)	29 (12.3%)	0.60
Other symptoms	107 (12.6%)	16 (7.1%)	55 (14.1%)	36 (15.3%)	0.015
Fully recovered from infection ^4^	713 (83.9%)	181 (80.8%)	343 (87.7%)	189 (80.4%)	0.019

Legend: ^1^ Adolescents: 12–17 years; older children: 6–11 years; young children: 0–5 years. ^2^ Pearson’s Chi-squared test. ^3^ N (%). ^4^ At enrolment. ^5^ The term “comorbidities” summarizes responses to the question investigating whether the participant had received a diagnosis or had been treated for a list of health problems occurring prior to COVID-19 infection and still ongoing. ^6^ In the first 14 days of COVID-19 infection.

## Data Availability

No new data were created or analyzed in this study. Data sharing is not applicable to this article. Data is contained within the article or supplementary material.

## Supplementary Materials

The following supporting information can be downloaded at: https://www.mdpi.com/article/10.3390/children13020279/s1, Figure S1: Scree plot indicating the contributions of each variable to Principal Components (Dim-1 and Dim-2) considering all enrolled patients; Figure S2: Scree plot indicating the contributions of each variable to Principal Components (Dim-1 and Dim-2) considering patients with PASC; Table S1: Variables characterizing each cluster determined considering all enrolled patients; Table S2: Variables characterizing each cluster determined considering patients with PASC.

## Abbreviations

The following abbreviations are used in this manuscript:

HCPC	Hierarchical Clustering on Principal Components
IQR	interquartile range
ISARIC	International Severe Acute Respiratory and Emerging Infection Consortium
