# Supplementary material for: Phenotyping Pediatric Long COVID: Symptom Clusters from a Longitudinal Multicenter Italian Cohort
Source: Children (Basel). 2026 Feb 18;13(2):279. doi: 10.3390/children13020279 (PMC12938949; doi:10.3390/children13020279)
Supplement: Supplementary file 1 [file children-13-00279-s001.zip › children-4117214-supplementary.pdf]

APPENDIX

Figure S1 – Scree plot indicating the contributions of each variable to Principal Components (Dim-1 and Dim-2) considering all enrolled patients

Figure S2 – Scree plot indicating the contributions of each variable to Principal Components (Dim-1 and Dim-2) considering patients with PASC

Table S1 – Variables characterizing each cluster determined considering all enrolled patients.

Table S2 – Variables characterizing each cluster determined considering patients with PASC.

Figure S1 – Scree plot indicating the contributions of each variable to Principal Components (Dim-1 and Dim-2) considering all enrolled patients

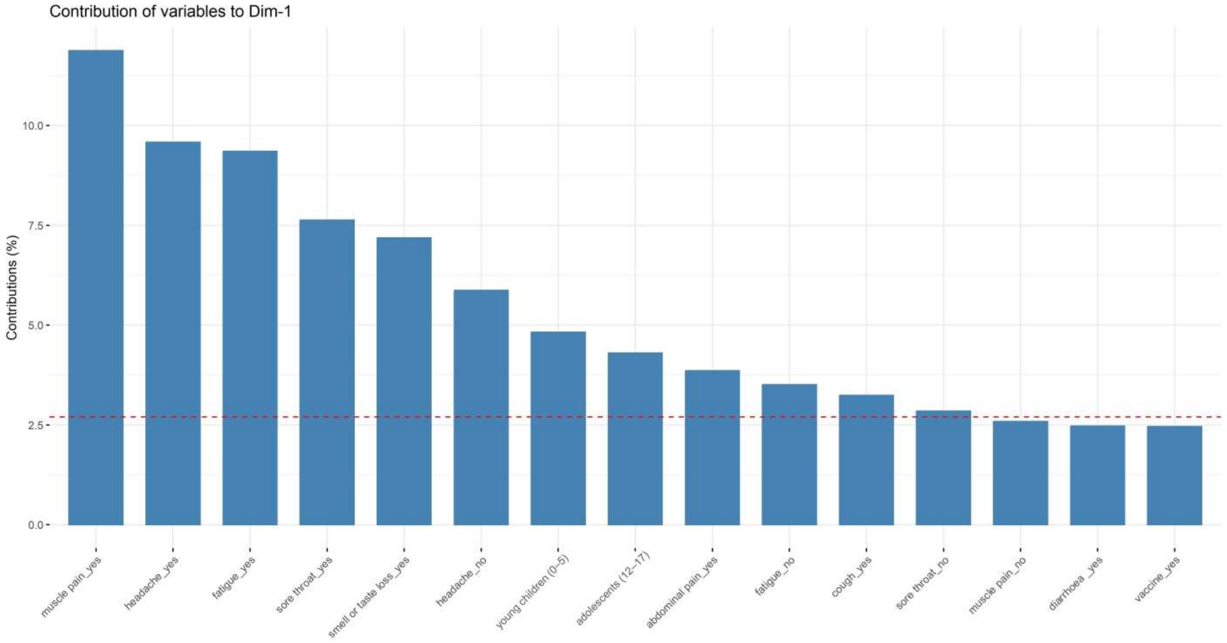

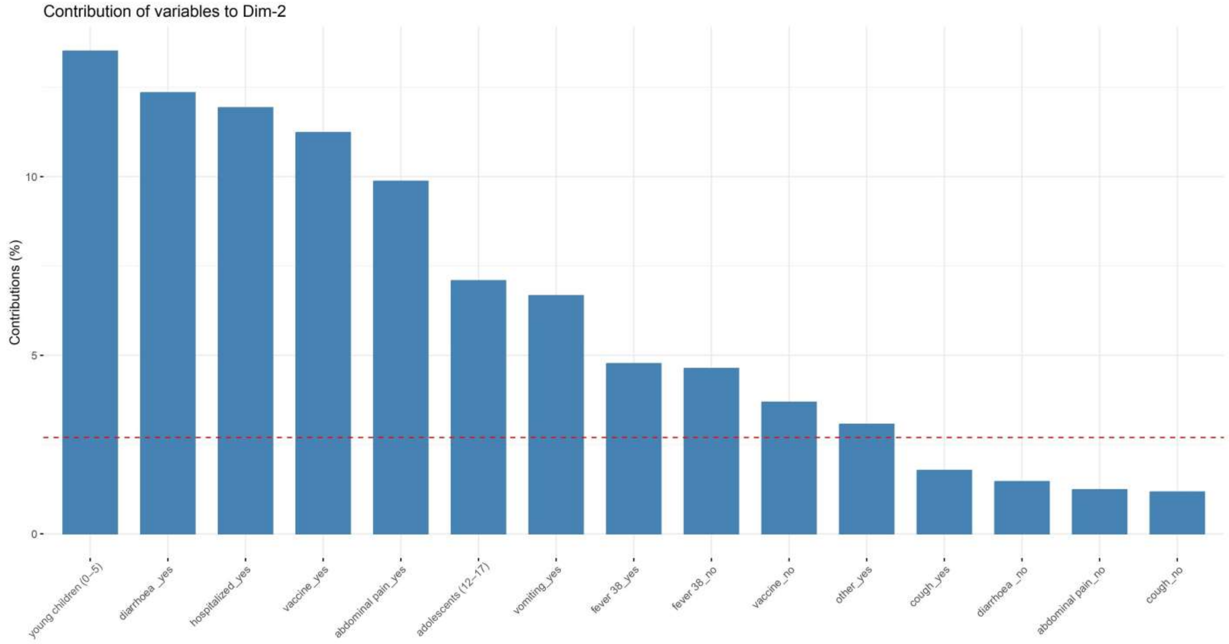

Legend: The bar plots display the percentage contribution of each variable to Dim-1 (top) and Dim-2 (bottom). The red dashed line indicates the expected average contribution if all variables contributed equally

Figure S2 – Scree plot indicating the contributions of each variable to Principal Components (Dim-1 and Dim-2) considering patients with PASC

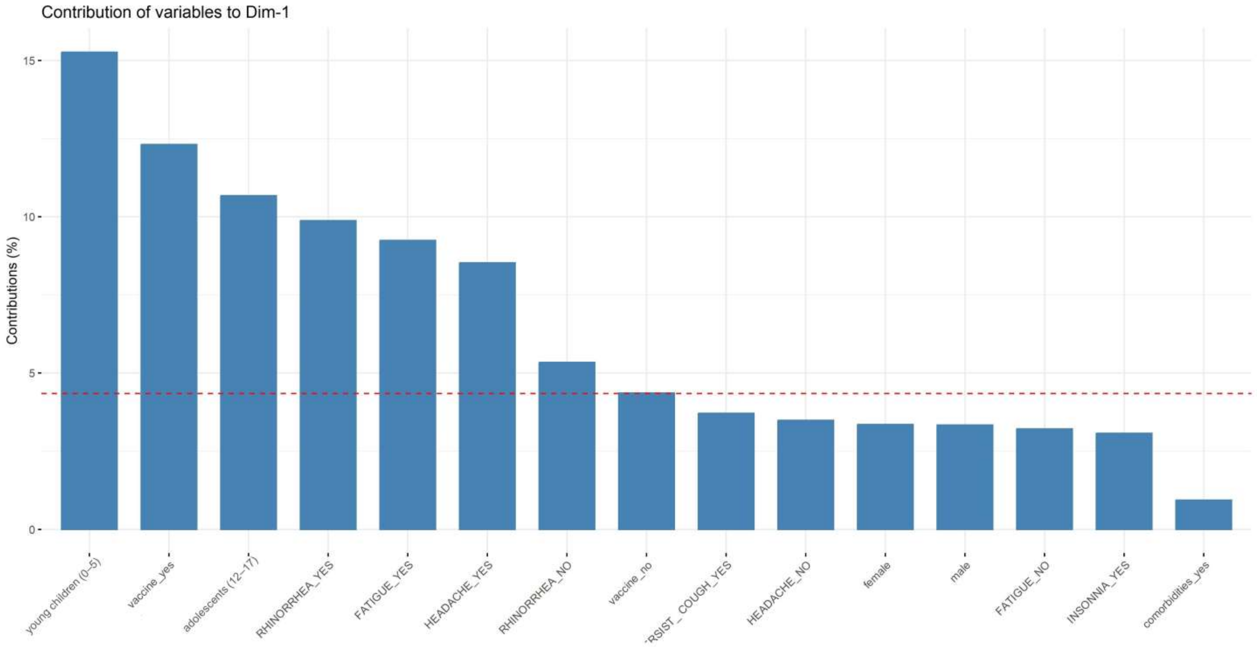

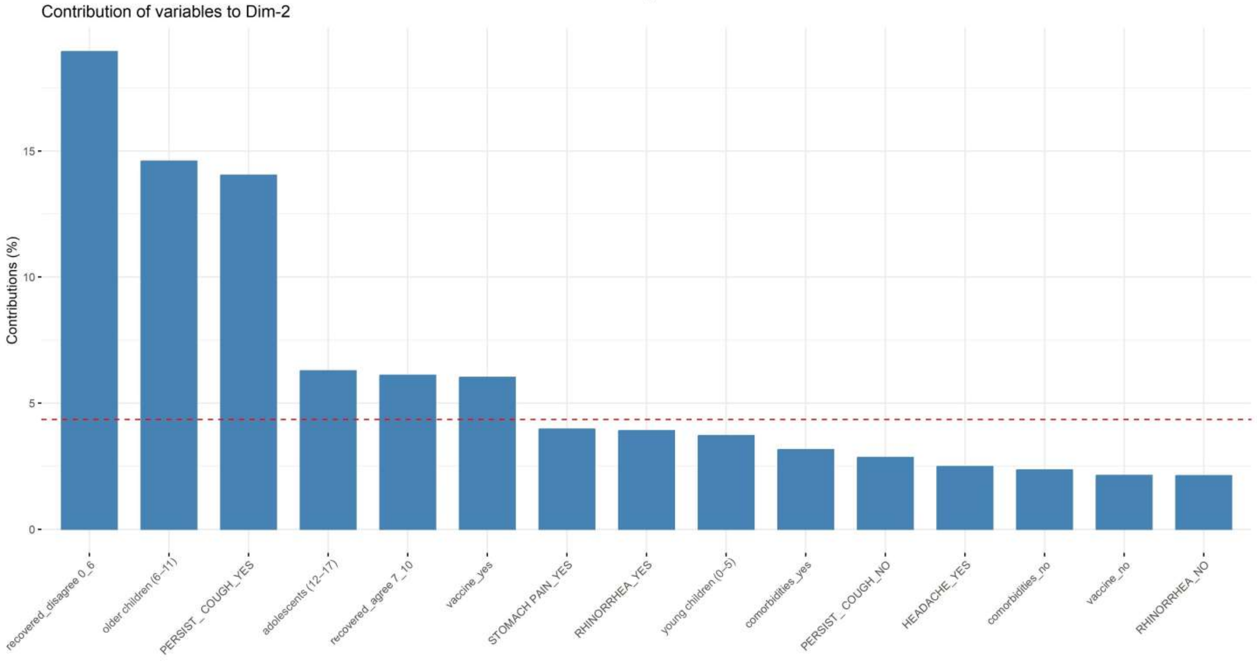

Legend: The bar plots display the percentage contribution of each variable to Dim-1 (top) and Dim-2 (bottom). The red dashed line indicates the expected average contribution if all variables contributed equally

Table S1 – Variables characterizing each cluster determined considering all enrolled patients.

Cluster 1					
	Cla/Mod	Mod/Cla	Global	p value	v test
age=young children (0–5)	88,51	91,63	27,65	2,40E-142	25,40
headache=headache_no	40,61	94,27	62,00	1,54E-37	12,80
vaccine=vaccine_no	35,16	99,12	75,29	2,44E-30	11,45
hospitalized=hospitalized_yes	90,57	21,15	6,24	4,69E-24	10,12

fatigue=fatigue_no	35,11	95,59	72,71	7,13E-24	10,07
muscle pain=muscle pain_no	31,38	96,48	82,12	1,75E-13	7,37
smell or taste loss=smell or taste loss_no	30,01	99,56	88,59	3,98E-13	7,26
RHINORRHEA=RHINORRHEA_YES	54,08	23,35	11,53	9,45E-10	6,12
fever 38=fever 38_yes	35,32	65,20	49,29	2,00E-08	5,61
HEADACHE=HEADACHE_NO	28,74	97,36	90,47	6,03E-06	4,53
FATIGUE=FATIGUE_NO	28,41	97,36	91,53	6,33E-05	4,00
PERSIST_ COUGH=PERSIST_COUGH_YES	53,19	11,01	5,53	8,26E-05	3,94
sore throat=sore throat_no	30,21	82,38	72,82	1,08E-04	3,87
comorbidities=comorbidities_no	31,10	73,57	63,18	1,24E-04	3,84
cough=cough_yes	31,56	47,14	39,88	9,64E-03	2,59
abdominal pain=abdominal pain_no	27,81	92,51	88,82	3,56E-02	2,10
current health=current_health_not at all	43,33	5,73	3,53	4,71E-02	1,99
abdominal pain=abdominal pain_yes	17,89	7,49	11,18	3,56E-02	-2,10
cough=cough_no	23,48	52,86	60,12	9,64E-03	-2,59
comorbidities=comorbidities_yes	19,17	26,43	36,82	1,24E-04	-3,84
sore throat=sore throat_yes	17,32	17,62	27,18	1,08E-04	-3,87
PERSIST_ COUGH=PERSIST_COUGH_NO	25,16	88,99	94,47	8,26E-05	-3,94
FATIGUE=FATIGUE_YES	8,33	2,64	8,47	6,33E-05	-4,00
HEADACHE=HEADACHE_YES	7,41	2,64	9,53	6,03E-06	-4,53
fever 38=fever 38_no	18,33	34,80	50,71	2,00E-08	-5,61
RHINORRHEA=RHINORRHEA_NO	23,14	76,65	88,47	9,45E-10	-6,12
smell or taste loss=smell or taste loss_yes	1,03	0,44	11,41	3,98E-13	-7,26
muscle pain=muscle pain_yes	5,26	3,52	17,88	1,75E-13	-7,37
fatigue=fatigue_yes	4,31	4,41	27,29	7,13E-24	-10,07
hospitalized=hospitalized_no	22,46	78,85	93,76	4,69E-24	-10,12
age=adolescents (12–17)	2,23	2,20	26,35	1,93E-28	-11,06
vaccine=vaccine_yes	0,95	0,88	24,71	2,44E-30	-11,45
headache=headache_yes	4,02	5,73	38,00	1,54E-37	-12,80
age=older children (6–11)	3,58	6,17	46,00	6,33E-52	-15,16

Cluster 2

	Cla/Mod	Mod/Cla	Global	p value	v test
age=older children (6–11)	70,84	69,60	46,00	2,00E-39	13,14
cough=cough_no	61,64	79,15	60,12	3,69E-27	10,79
fever 38=fever 38_no	64,04	69,35	50,71	7,91E-25	10,29
muscle pain=muscle pain_no	53,87	94,47	82,12	3,20E-20	9,21
diarrhoea=diarrhoea_no	51,32	97,99	89,41	3,39E-16	8,16
sore throat=sore throat_no	54,77	85,18	72,82	1,21E-14	7,71
hospitalized=hospitalized_no	49,81	99,75	93,76	3,48E-14	7,58
abdominal pain=abdominal pain_no	50,73	96,23	88,82	2,16E-11	6,69
PASC=PASC_NO	54,64	78,39	67,18	4,60E-11	6,58
RHINORRHEA=RHINORRHEA_NO	50,40	95,23	88,47	2,51E-09	5,96
runny nose=runny nose_no	56,14	64,32	53,65	4,46E-09	5,87
recovered=recovered_agree 7_10	50,91	91,21	83,88	3,03E-08	5,54
smell or taste loss=smell or taste loss_no	49,93	94,47	88,59	2,29E-07	5,17
vomiting=vomiting_no	49,80	94,97	89,29	2,70E-07	5,14

fatigue=fatigue_no	52,10	80,90	72,71	4,10E-07	5,06
vaccine=vaccine_yes	60,00	31,66	24,71	1,09E-05	4,40
PERSIST_ COUGH=PERSIST_COUGH_NO	48,57	97,99	94,47	1,39E-05	4,35
headache=headache_no	52,56	69,60	62,00	1,81E-05	4,29
current health=current_health_complete	53,81	55,03	47,88	9,33E-05	3,91
POOR APPETITE=POOR APPETITE_NO	47,79	97,74	95,76	6,93E-03	2,70
INSONNIA=INSONNIA_NO	47,78	97,24	95,29	1,18E-02	2,52
other=other_no	48,45	90,45	87,41	1,20E-02	2,51
FATIGUE=FATIGUE_NO	48,07	93,97	91,53	1,63E-02	2,40
HEADACHE=HEADACHE_NO	48,11	92,96	90,47	2,00E-02	2,33
HEADACHE=HEADACHE_YES	34,57	7,04	9,53	2,00E-02	-2,33
FATIGUE=FATIGUE_YES	33,33	6,03	8,47	1,63E-02	-2,40
other=other_yes	35,51	9,55	12,59	1,20E-02	-2,51
INSONNIA=INSONNIA_YES	27,50	2,76	4,71	1,18E-02	-2,52
current health=current_health_not at all	23,33	1,76	3,53	8,21E-03	-2,64
POOR APPETITE=POOR APPETITE_YES	25,00	2,26	4,24	6,93E-03	-2,70
current health=current_health_partial	41,65	43,22	48,59	3,32E-03	-2,94
headache=headache_yes	37,46	30,40	38,00	1,81E-05	-4,29
PERSIST_ COUGH=PERSIST_COUGH_YES	17,02	2,01	5,53	1,39E-05	-4,35
vaccine=vaccine_no	42,50	68,34	75,29	1,09E-05	-4,40
fatigue=fatigue_yes	32,76	19,10	27,29	4,10E-07	-5,06
vomiting=vomiting_yes	21,98	5,03	10,71	2,70E-07	-5,14
smell or taste loss=smell or taste loss_yes	22,68	5,53	11,41	2,29E-07	-5,17
recovered=recovered_disagree 0_6	25,55	8,79	16,12	3,03E-08	-5,54
runny nose=runny nose_yes	36,04	35,68	46,35	4,46E-09	-5,87
RHINORRHEA=RHINORRHEA_YES	19,39	4,77	11,53	2,51E-09	-5,96
PASC=PASC_YES	30,82	21,61	32,82	4,60E-11	-6,58
abdominal pain=abdominal pain_yes	15,79	3,77	11,18	2,16E-11	-6,69
hospitalized=hospitalized_yes	1,89	0,25	6,24	3,48E-14	-7,58
sore throat=sore throat_yes	25,54	14,82	27,18	1,21E-14	-7,71
diarrhoea=diarrhoea_yes	8,89	2,01	10,59	3,39E-16	-8,16
muscle pain=muscle pain_yes	14,47	5,53	17,88	3,20E-20	-9,21
fever 38=fever 38_yes	29,12	30,65	49,29	7,91E-25	-10,29
cough=cough_yes	24,48	20,85	39,88	3,69E-27	-10,79
age=young children (0–5)	5,11	3,02	27,65	9,18E-60	-16,30

Cluster 3					
	Cla/Mod	Mod/Cla	Global	p value	v test
headache=headache_yes	58,51	84,00	38,00	8,69E-63	16,72
muscle pain=muscle pain_yes	80,26	54,22	17,88	2,42E-55	15,67
fatigue=fatigue_yes	62,93	64,89	27,29	4,79E-46	14,25
sore throat=sore throat_yes	57,14	58,67	27,18	8,29E-33	11,93
smell or taste loss=smell or taste loss_yes	76,29	32,89	11,41	5,37E-28	10,97
cough=cough_yes	43,95	66,22	39,88	9,66E-21	9,34
abdominal pain=abdominal pain_yes	66,32	28,00	11,18	4,16E-18	8,67
age=adolescents (12–17)	49,11	48,89	26,35	6,30E-18	8,63
diarrhoea=diarrhoea	62,22	24,89	10,59	4,03E-14	7,56

runny nose=runny nose_yes	38,32	67,11	46,35	3,00E-13	7,29
HEADACHE=HEADACHE_YES	58,02	20,89	9,53	2,89E-10	6,30
FATIGUE=FATIGUE_YES	58,33	18,67	8,47	2,59E-09	5,96
fever 38=fever 38_yes	35,56	66,22	49,29	2,80E-09	5,94
PASC=PASC_YES	39,43	48,89	32,82	4,46E-09	5,87
current health=current_health_partial	33,90	62,22	48,59	1,82E-06	4,77
comorbidities=comorbidities_yes	35,78	49,78	36,82	3,53E-06	4,64
vaccine=vaccine_yes	39,05	36,44	24,71	3,67E-06	4,63
recovered=recovered_disagree 0_6	42,34	25,78	16,12	1,03E-05	4,41
vomiting=vomiting_yes	42,86	17,33	10,71	3,45E-04	3,58
hospitalized=hospitalized_no	27,73	98,22	93,76	4,51E-04	3,51
other=other_yes	40,19	19,11	12,59	9,43E-04	3,31
INSONNIA=INSONNIA_YES	50,00	8,89	4,71	1,24E-03	3,23
POOR APPETITE=POOR APPETITE_YES	47,22	7,56	4,24	6,80E-03	2,71
sex=female	29,58	53,78	48,12	4,82E-02	1,98
sex=male	23,58	46,22	51,88	4,82E-02	-1,98
POOR APPETITE=POOR APPETITE_NO	25,55	92,44	95,76	6,80E-03	-2,71
INSONNIA=INSONNIA_NO	25,31	91,11	95,29	1,24E-03	-3,23
other=other_no	24,50	80,89	87,41	9,43E-04	-3,31
hospitalized=hospitalized_yes	7,55	1,78	6,24	4,51E-04	-3,51
vomiting=vomiting_no	24,51	82,67	89,29	3,45E-04	-3,58
recovered=recovered_agree 7_10	23,42	74,22	83,88	1,03E-05	-4,41
vaccine=vaccine_no	22,34	63,56	75,29	3,67E-06	-4,63
comorbidities=comorbidities_no	21,04	50,22	63,18	3,53E-06	-4,64
current health=current_health_complete	18,43	33,33	47,88	3,05E-07	-5,12
PASC=PASC_NO	20,14	51,11	67,18	4,46E-09	-5,87
fever 38=fever 38_no	17,63	33,78	50,71	2,80E-09	-5,94
FATIGUE=FATIGUE_NO	23,52	81,33	91,53	2,59E-09	-5,96
HEADACHE=HEADACHE_NO	23,15	79,11	90,47	2,89E-10	-6,30
runny nose=runny nose_no	16,23	32,89	53,65	3,00E-13	-7,29
diarrhoea=diarrhoea_no	22,24	75,11	89,41	4,03E-14	-7,56
abdominal pain=abdominal pain_no	21,46	72,00	88,82	4,16E-18	-8,67
age=young children (0–5)	6,38	6,67	27,65	3,43E-19	-8,95
cough=cough_no	14,87	33,78	60,12	9,66E-21	-9,34
smell or taste loss=smell or taste loss_no	20,05	67,11	88,59	5,37E-28	-10,97
sore throat=sore throat_no	15,02	41,33	72,82	8,29E-33	-11,93
fatigue=fatigue_no	12,78	35,11	72,71	4,79E-46	-14,25
muscle pain=muscle pain_no	14,76	45,78	82,12	2,42E-55	-15,67
headache=headache_no	6,83	16,00	62,00	8,69E-63	-16,72

Legend: Cla/Mod represents the percentage of patients that have the modality "i" and belong to the cluster "j" and Mod/Cla, the percentage of patients belonging to cluster "j" that show the modality "i". The variables in uppercase indicate post-acute sequelae, while those in lowercase are acute symptoms and patient characteristics. V-test values indicate standardized differences between cluster-specific and overall proportions; positive values denote over-representation within a cluster and negative values under-representation.

Table S2 – Variables characterizing each cluster determined considering patients with PASC.

Cluster 1					
	Cla/Mod	Mod/Cla	Global	p value	v test
age=young children (0–5)	90,70	88,64	30,82	4,79E-48	14,56
RHINORRHEA=RHINORRHEA_YES	64,29	71,59	35,13	9,83E-18	8,58
HEADACHE=HEADACHE_NO	42,93	96,59	70,97	1,70E-12	7,06
headache=headache_no	47,74	84,09	55,56	2,04E-11	6,70
PERSIST_ COUGH=PERSIST_COUGH_YES	74,47	39,77	16,85	3,17E-11	6,64
FATIGUE=FATIGUE_NO	41,06	96,59	74,19	1,60E-10	6,40
vaccine=vaccine_no	40,78	95,45	73,84	1,25E-09	6,07
fatigue=fatigue_no	43,68	86,36	62,37	5,57E-09	5,83
muscle pain=muscle pain_no	39,13	92,05	74,19	9,78E-07	4,90
hospitalized=hospitalized_yes	82,35	15,91	6,09	1,33E-05	4,35
smell or taste loss=smell or taste loss_no	35,93	94,32	82,80	2,56E-04	3,66
recovered=recovered_disagree 0_6	42,65	32,95	24,37	2,69E-02	2,21
fever 38=fever 38_yes	37,16	62,50	53,05	3,26E-02	2,14
sex=male	37,14	59,09	50,18	4,47E-02	2,01
cough=cough_yes	37,31	56,82	48,03	4,78E-02	1,98
cough=cough_no	26,21	43,18	51,97	4,78E-02	-1,98
sex=female	25,90	40,91	49,82	4,47E-02	-2,01
fever 38=fever 38_no	25,19	37,50	46,95	3,26E-02	-2,14
recovered=recovered_agree 7_10	27,96	67,05	75,63	2,69E-02	-2,21
smell or taste loss=smell or taste loss_yes	10,42	5,68	17,20	2,56E-04	-3,66
hospitalized=hospitalized_no	28,24	84,09	93,91	1,33E-05	-4,35
muscle pain=muscle pain_yes	9,72	7,95	25,81	9,78E-07	-4,90
fatigue=fatigue_yes	11,43	13,64	37,63	5,57E-09	-5,83
vaccine=vaccine_yes	5,48	4,55	26,16	1,25E-09	-6,07
FATIGUE=FATIGUE_YES	4,17	3,41	25,81	1,60E-10	-6,40
PERSIST_ COUGH=PERSIST_COUGH_NO	22,84	60,23	83,15	3,17E-11	-6,64
headache=headache_yes	11,29	15,91	44,44	2,04E-11	-6,70
HEADACHE=HEADACHE_YES	3,70	3,41	29,03	1,70E-12	-7,06
age=adolescents (12–17)	2,47	2,27	29,03	8,19E-14	-7,47
age=older children (6–11)	7,14	9,09	40,14	3,19E-14	-7,59
RHINORRHEA=RHINORRHEA_NO	13,81	28,41	64,87	9,83E-18	-8,58

Cluster 2					
	Cla/Mod	Mod/Cla	Global	p value	v test
age=older children (6–11)	84,82	77,87	40,14	1,14E-31	11,71
recovered=recovered_agree 7_10	52,61	90,98	75,63	5,62E-08	5,43
vaccine=vaccine_no	52,91	89,34	73,84	9,75E-08	5,33
PERSIST_ COUGH=PERSIST_COUGH_NO	50,43	95,90	83,15	1,33E-07	5,28
RHINORRHEA=RHINORRHEA_NO	53,59	79,51	64,87	5,35E-06	4,55
HEADACHE=HEADACHE_YES	59,26	39,34	29,03	9,27E-04	3,31
STOMACH PAIN=STOMACH PAIN_YES	70,00	17,21	10,75	2,54E-03	3,02
sex=male	52,14	59,84	50,18	4,66E-03	2,83

INSONNIA=INSONNIA_NO	46,86	91,80	85,66	9,46E-03	2,59
headache=headache_yes	51,61	52,46	44,44	1,83E-02	2,36
cough=cough_no	50,34	59,84	51,97	2,12E-02	2,31
hospitalized=hospitalized_no	45,42	97,54	93,91	2,49E-02	2,24
runny nose=runny nose_no	50,78	53,28	45,88	2,99E-02	2,17
current health=current_health_complete	53,66	36,07	29,39	3,28E-02	2,14
runny nose=runny nose_yes	37,75	46,72	54,12	2,99E-02	-2,17
hospitalized=hospitalized_yes	17,65	2,46	6,09	2,49E-02	-2,24
cough=cough_yes	36,57	40,16	48,03	2,12E-02	-2,31
headache=headache_no	37,42	47,54	55,56	1,83E-02	-2,36
INSONNIA=INSONNIA_YES	25,00	8,20	14,34	9,46E-03	-2,59
sex=female	35,25	40,16	49,82	4,66E-03	-2,83
STOMACH PAIN=STOMACH PAIN_NO	40,56	82,79	89,25	2,54E-03	-3,02
HEADACHE=HEADACHE_NO	37,37	60,66	70,97	9,27E-04	-3,31
current health=current_health_not at all	0,00	0,00	5,02	2,45E-04	-3,67
age=adolescents (12–17)	23,46	15,57	29,03	9,81E-06	-4,42
RHINORRHEA=RHINORRHEA_YES	25,51	20,49	35,13	5,35E-06	-4,55
PERSIST_ COUGH=PERSIST_COUGH_YES	10,64	4,10	16,85	1,33E-07	-5,28
vaccine=vaccine_yes	17,81	10,66	26,16	9,75E-08	-5,33
recovered=recovered_disagree 0_6	16,18	9,02	24,37	5,62E-08	-5,43
age=young children (0–5)	9,30	6,56	30,82	3,55E-16	-8,15

Cluster 3					
	Cla/Mod	Mod/Cla	Global	p value	v test
age=adolescents (12–17)	74,07	86,96	29,03	4,56E-33	11,98
vaccine=vaccine_yes	76,71	81,16	26,16	7,30E-31	11,55
sex=female	38,85	78,26	49,82	3,52E-08	5,51
muscle pain=muscle pain_yes	48,61	50,72	25,81	2,06E-07	5,19
FATIGUE=FATIGUE_YES	48,61	50,72	25,81	2,06E-07	5,19
RHINORRHEA=RHINORRHEA_NO	32,60	85,51	64,87	1,76E-05	4,29
headache=headache_yes	37,10	66,67	44,44	2,14E-05	4,25
fatigue=fatigue_yes	39,05	59,42	37,63	2,47E-05	4,22
INSONNIA=INSONNIA_YES	50,00	28,99	14,34	1,94E-04	3,73
recovered=recovered_disagree 0_6	41,18	40,58	24,37	5,36E-04	3,46
current health=current_health_not at all	64,29	13,04	5,02	1,71E-03	3,14
runny nose=runny nose_yes	31,79	69,57	54,12	2,97E-03	2,97
HEADACHE=HEADACHE_YES	37,04	43,48	29,03	3,15E-03	2,95
sore throat=sore throat_yes	35,48	47,83	33,33	4,09E-03	2,87
smell or taste loss=smell or taste loss_yes	41,67	28,99	17,20	4,57E-03	2,84
hospitalized=hospitalized_no	26,34	100,00	93,91	6,75E-03	2,71
comorbidities=comorbidities_yes	31,09	53,62	42,65	3,60E-02	2,10
current health=current_health_partial	28,42	75,36	65,59	4,82E-02	1,98
comorbidities=comorbidities_no	20,00	46,38	57,35	3,60E-02	-2,10
hospitalized=hospitalized_yes	0,00	0,00	6,09	6,75E-03	-2,71
smell or taste loss=smell or taste loss_no	21,21	71,01	82,80	4,57E-03	-2,84
sore throat=sore throat_no	19,35	52,17	66,67	4,09E-03	-2,87
HEADACHE=HEADACHE_NO	19,70	56,52	70,97	3,15E-03	-2,95

runny nose=runny nose_no	16,41	30,43	45,88	2,97E-03	-2,97
recovered=recovered_agree 7_10	19,43	59,42	75,63	5,36E-04	-3,46
INSONNIA=INSONNIA_NO	20,50	71,01	85,66	1,94E-04	-3,73
current health=current_health_complete	9,76	11,59	29,39	9,16E-05	-3,91
fatigue=fatigue_no	16,09	40,58	62,37	2,47E-05	-4,22
headache=headache_no	14,84	33,33	55,56	2,14E-05	-4,25
RHINORRHEA=RHINORRHEA_YES	10,20	14,49	35,13	1,76E-05	-4,29
muscle pain=muscle pain_no	16,43	49,28	74,19	2,06E-07	-5,19
FATIGUE=FATIGUE_NO	16,43	49,28	74,19	2,06E-07	-5,19
sex=male	10,71	21,74	50,18	3,52E-08	-5,51
age=older children (6–11)	8,04	13,04	40,14	3,30E-08	-5,52
age=young children (0–5)	0,00	0,00	30,82	8,04E-14	-7,47
vaccine=vaccine_no	6,31	18,84	73,84	7,30E-31	-11,55

Legend: Cla/Mod represents the percentage of patients that have the modality “i” and belong to the cluster “j” and Mod/Cla, the percentage of patients belonging to cluster “j” that show the modality “i”. The variables in uppercase indicate post-acute sequelae, while those in lowercase are acute symptoms and patient characteristics. V-test values indicate standardized differences between cluster-specific and overall proportions; positive values denote over-representation within a cluster and negative values under-representation.
